# Quadratic convergence of monotone iterates for semilinear elliptic obstacle problems

**DOI:** 10.1186/s13660-017-1513-x

**Published:** 2017-09-25

**Authors:** Jinping Zeng, Haowen Chen, Hongru Xu

**Affiliations:** 1 College of Computer Science, Dongguan University of Technology, Dongguan, Guangdong 523808 P.R. China; 2 College of Mathematics and Econometrics, Hunan University, Changsha, 410082 P.R. China; 3 School of Mathematics, Jiaying University, Meizhou, Guangdong 514015 P.R. China

**Keywords:** complementarity problem, obstacle problem, upper and lower solution, monotone iteration, quadratic convergence, 65F10, 65N30, 90C33

## Abstract

In this paper, we consider the numerical solution for the discretization of semilinear elliptic complementarity problems. A monotone algorithm is established based on the upper and lower solutions of the problem. It is proved that iterates, generated by the algorithm, are a pair of upper and lower solution iterates and converge monotonically from above and below, respectively, to the solution of the problem. Moreover, we investigate the convergence rate for the monotone algorithm and prove quadratic convergence of the algorithm. The monotone and quadratic convergence results are also extended to the discrete problems of the two-sided obstacle problems with a semilinear elliptic operator. We also present some simple numerical experiments.

## Introduction

In this paper, we consider the following semilinear elliptic complementarity problem of finding ${\mathcal {U}}\in{ \mathcal {K}}=\{{\mathcal {V}}\in H_{0}^{1}(\Omega):{\mathcal {V}}\ge{ \mathcal {\varphi}}, \mbox{ a.e. in } \Omega\}$ such that

1.1 $$ a({\mathcal {U}},{\mathcal {V}}-{\mathcal {U}})+\bigl({\mathcal {F}}({\mathcal {U}},\cdot),{\mathcal {V}-U}\bigr)\ge 0, \quad \forall{ \mathcal {V}}\in{ \mathcal {K}}, $$

where $\Omega\in R^{2}$ is a bounded convex polygonal with boundary *∂*Ω, ${\mathcal {F}}({\mathcal {V}},x)$ is continuously differentiable in variable ${\mathcal {V}}$ with $\frac{ \partial{ \mathcal {F}}}{ \partial{ \mathcal {V}}}\geq C^{*}\ge0$ on ${\mathcal {K}}\times\bar{\Omega}$, $\varphi\in H^{2}(\Omega)$ with $\varphi|_{\partial\Omega}\leq0$, and

$$a({\mathcal {U}},{\mathcal {V}})= \int_{\Omega}\nabla{ \mathcal {U}}\nabla{ \mathcal {V}} \,dx+ \int _{\Omega}\bigl[(\vec{\beta}\cdot\nabla{ \mathcal {U}}){\mathcal {V}} +\gamma{ \mathcal {U}V}\bigr]\,dx. $$

Here, $\vec{\beta}=(\beta_{1},\beta_{2})\in(L^{\infty}(\Omega))^{2}$, $\gamma \in L^{\infty}(\Omega)$ and $\gamma\ge0$ on Ω.

Problem (1.1) has been widely applied in many scientific, engineering or economic problems, e.g., in the diffusion problems involving Michaelis-Menten or second order irreversible reactions [1–5].

To solve problem (1.1), we generally apply a finite difference or finite element approximation to obtain a discrete problem. If we use standard finite difference or lumping finite element approximation with a Delaunay triangle triangulation (see, e.g., in [6–8]), the discrete problem can be a finite dimensional nonlinear complementarity problem of finding ${ u}\in K=\{{ v}\in R^{n}:{ v}\ge\phi\}$ such that

1.2 $$ A{ u}+{f}({ u})\ge0,\qquad ({ u}-\phi)^{T}\bigl(A{u}+{f}({u}) \bigr)=0, $$

where $A=(a_{ij})\in R^{n\times n}$ is an *M*-matrix, ${f}:R^{n}\rightarrow R^{n}$ is a diagonal and nondecreasing monotone mapping. In other words, matrix *A* has a nonnegative inverse $A^{-1}$ as well as non-positive off-diagonals, and mapping *f* has a form of $f(v)=(f_{i}(v_{i}))\in R^{n}$ with

1.3 $$ f'_{i}(v_{i})\geq c_{i}^{*}\geq0. $$

In this paper, we also assume that *A* is a positive definite matrix. That is, there exists a positive constant *σ* such that, for any vector $v\in R^{n}$,

1.4 $$ v^{T}Av\ge\sigma v^{T}v. $$

These conditions can be satisfied for suitable small meshsize, see, e.g., in [6, 7, 9].

The numerical algorithms for solving problem (1.2) have been developing rapidly. Among these algorithms, there are a kind of monotone algorithms based on the upper or lower solutions of the problem. We refer to [7, 8, 10, 11] and the references therein for details. These algorithms can be regarded as extensions of the monotone algorithms for solving elliptic boundary value problems or their discretizations (see, e.g., in [6, 12–14]). In these algorithms, any generated iterate is an upper (or lower) solution sequence which then converges to the solution monotonically. In this paper, we extend the monotone iterative approach for elliptic boundary value equation, presented in [6], to complementarity as well as two-sided obstacle problems. By using a pair of upper and lower solutions as two initial iterates, one can construct two monotone sequences which converge monotonically from above and below, respectively, to the solutions of the problems. Especially, the initial iterative solutions in the monotone iterative algorithms can be obtained directly by solving two discrete linear complementarity problems without any knowledge of the exact solution. Quadratic convergence is also proved for the algorithms.

The structure of the paper is as follows. In Section 2, we provide two procedures, in which only a pair of linear complementarity problems are needed to be solved. Following these procedures, we can obtain a pair of upper and lower solutions of the nonlinear complementarity problem we are concerned with. In Sections 3 and 4, we propose a monotone iterative algorithm and deal with the quadratic convergence of the monotone iterates, respectively. In Section 5, we extend the results obtained in Sections 2-4 to the two-sided obstacle problem. In Section 6, we present some simple numerical experiments.

## Upper and lower solutions and their initializations

The approach presented in this paper is based on the upper and lower solutions of the problems. In this section, we introduce the definitions of upper and lower solutions of problem (1.2) and discuss their properties.

Nonlinear complementarity problem (1.2) is equivalent to the following system of nonsmooth equations:

2.1 $$ \operatorname{min}\bigl\{ Au+f(u),u-\phi\bigr\} =0. $$

According to (2.1), let

$$S_{1}=\bigl\{ v\in R^{n}: \operatorname{min}\bigl\{ Av+f(v),v-\phi\bigr\} \geq0\bigr\} $$

and

$$S_{-1}=\bigl\{ v\in R^{n}: \operatorname{min}\bigl\{ Av+f(v),v-\phi\bigr\} \leq0\bigr\} . $$

Then $S_{1}$ and $S_{-1}$ are called the upper and lower solution sets of problem (1.2), respectively. Besides, any element of $S_{1}$ ($S_{-1}$) is called an upper (lower) solution of problem (1.2). It is well known that the solution of the problem is the minimal (maximal) element of upper (lower) solution set (see, e.g., in [9, 15, 16]).

It is obvious that $v\in S_{1}$ is equivalent to

$$Av+f(v)\geq0,\quad v\ge\phi, $$

that is, $v\in K$ and $Av+f(v)\geq0$. On the other hand, $v\in S_{-1}$ is equivalent to $(Av+f(v))_{i}\leq0$ holds for each index *i* satisfying $v_{i}>\phi_{i}$. Therefore, a lower solution *v* may not belong to a feasible set *K*. For instance, any *v* satisfying $Av+f(v)\le0$ is in $S_{-1}$. Let

$${ \tilde{S}}_{-1}=\bigl\{ v\in K: \operatorname{min}\bigl\{ Av+f(v),v-\phi\bigr\} \leq0 \bigr\} . $$

That is, $\tilde{S}_{-1}$ consists of lower solutions in *K*. Since the solution of problem (1.2) is in *K*, in the sequel, we will consider the lower solution in ${\tilde{S}}_{-1}$. Obviously, *ϕ* is a candidate of such lower solutions.

### Lemma 2.1

*For any* $w\in R^{n}$, *let* *ū* *be a solution of the following linear complementarity problem* (*LCP*) *of finding* $\bar{u}\in K$ *such that*

2.2 $$ \bigl(A+\Lambda^{*}\bigr)\bar{u}\ge\bar{g},\qquad (\bar{u}- \phi)^{T}\bigl[\bigl(A+\Lambda^{*}\bigr)\bar{u}-\bar{g}\bigr]=0, $$

*where* $\Lambda^{*}=\operatorname{diag}(c^{*}_{1},c^{*}_{2},\ldots,c^{*}_{n})\in R^{n\times n}$ *is a nonnegative diagonal matrix and*

$$\bar{g}= \bigl\vert Aw+f(w) \bigr\vert +\bigl(A+\Lambda^{*}\bigr)w. $$

*Then* $\bar{u}\in S_{1}$.

### Proof

Assuming that *ū* satisfies (2.2) and $\vert Aw+f(w) \vert +Aw+f(w)\ge0$,

2.3 $$\begin{aligned} A\bar{u}+f(\bar{u}) ={}&\bigl(A+\Lambda^{*}\bigr)\bar{u}-\bar{g} \\ &{}+ \bigl\vert Aw+f(w) \bigr\vert +Aw+f(w) \\ &{}+f(\bar{u})-f(w)+\Lambda^{*}(w-\bar{u}) \\ \ge{}& \int_{0}^{1}\bigl[f'(w+tz)-\Lambda^{*} \bigr]z\,dt, \end{aligned}$$

where $z=\bar{u}-w$. It follows from (2.2) again that

2.4 $$ \bigl(A+\Lambda^{*}\bigr)z\geq \bigl\vert Aw+f(w) \bigr\vert \geq0. $$

Since *A* is an *M*-matrix, so is $A+\Lambda^{*}$ (see, e.g., in [13, 14]). That is, $(A+\Lambda^{*})^{-1}\geq0$. This together with (2.4) implies $z\geq0$, and hence by (1.3), $\int_{0}^{1}[f'(w+tz)-\Lambda^{*}]z\,dt\ge0$. Therefore, by (2.3), we get

$$A\bar{u}+f(\bar{u})\geq0, $$

which together with $\bar{u} \in K$ implies $\bar{u}\in S_{1}$. □

For the lower solution, we have a similar result as follows.

### Lemma 2.2

*For any* $w\in R^{n}$, *let* $\underline{u}$ *be a solution of the following LCP of finding* $\underline{u}\in K$ *such that*

2.5 $$ \bigl(A+\Lambda^{*}\bigr)\underline{u}\leq\underline{g},\qquad (\underline{u}-\phi )^{T}\bigl[\bigl(A+\Lambda^{*}\bigr)\underline{u}-\underline{g} \bigr]=0, $$

*where*

$$\underline{g}=- \bigl\vert Aw+f(w) \bigr\vert +\bigl(A+\Lambda^{*}\bigr)w. $$

*Then* $\underline{u}\in\tilde{S}_{-1}$.

Problems (2.2) and (2.5) can be regarded as lower and upper obstacle problems with variables *ū* and $-\underline{u}$, respectively. According to Lemmas 2.1 and 2.2, we can obtain a pair of upper and lower solutions of nonlinear complementarity problem (1.2) by solving two linear complementarity problems (2.2) and (2.5). As for linear complementarity problems, there are many classic and efficient iterative or direct algorithms to solve them. We refer to [11, 15–17] for further discussions.

## Monotone iterative algorithm for complementarity problem

In this section, we propose an algorithm for solving the nonlinear complementarity problem (1.2) and discuss its monotone convergence. Firstly, we present the algorithm as follows.

### Algorithm 3.1

Let the initials $u^{(0)}_{1}\in S_{1}$ and $u^{(0)}_{-1}\in{\tilde{S}}_{-1}$. For $k\ge1$, we calculate a pair of iterates by solving the following linear complementarity problems of finding $u_{\alpha}^{(k)}\in K$, $\alpha=1, -1$, respectively, such that

3.1 $$ \textstyle\begin{cases} A^{(k)}(u_{\alpha}^{(k)}-u_{\alpha}^{(k-1)})+r(u_{\alpha}^{(k-1)})\ge 0,\\ (u_{\alpha}^{(k)}-\phi)^{T}[A^{(k)}(u_{\alpha}^{(k)}-u_{\alpha }^{(k-1)})+r(u_{\alpha}^{(k-1)})]=0, \end{cases} $$

where

3.2 $$ r\bigl(u_{\alpha}^{(k-1)}\bigr)=Au_{\alpha}^{(k-1)}+f \bigl(u_{\alpha}^{(k-1)}\bigr), \quad \alpha=1,-1, $$

$A^{(k)}=A+\Lambda^{(k-1)}$, and $\Lambda^{(k-1)}$ is a diagonal matrix with diagonals

3.3 $$ \lambda_{i}^{(k-1)}=\max\bigl\{ f'_{i}(v_{i}):\bigl(u^{(k-1)}_{-1} \bigr)_{i}\leq v_{i}\leq \bigl(u_{1}^{(k-1)} \bigr)_{i}\bigr\} \ge c_{i}^{*},\quad i=1,2,\ldots, n. $$

Direct algorithms with a polynomial computational complexity are fruitful for subproblems (3.1). Especially, there are a lot of polynomial algorithms for linear complementarity problems with an *M*-matrix. We refer to [15–19] for more details.

By Lemmas 2.1 and 2.2, for any $w\in R^{n}$, *ū* and $\underline{u}$, generated by (2.2) and (2.5), are in $S_{1}$ and ${\tilde{S}} _{-1}$, respectively. So, we may let the initials in Algorithm 3.1 be obtained by solving (2.2) and (2.5). That is,

$$u^{(0)}_{1}=\bar{u} \quad\mbox{and}\quad u^{(0)}_{-1}= \underline{u}. $$

### Remark 3.1

Noting that *A* is an *M*-matrix and $\Lambda^{(k-1)}$ is a diagonal matrix with nonnegative diagonals, $A^{(k)}$ is also an *M*-matrix. Therefore, similar to $S_{1}$ and ${\tilde{S}}_{-1}$, we can define the upper and lower solution sets of problem (3.1), respectively, as follows:

3.4 $$\begin{aligned} &S_{1}^{(k,\alpha)}=\bigl\{ v\in R^{n}: \operatorname{min} \bigl\{ A^{(k)}\bigl(v-u_{\alpha }^{(k-1)}\bigr)+r \bigl(u_{\alpha}^{(k-1)}\bigr),v-\phi\bigr\} \geq0\bigr\} , \end{aligned}$$

3.5 $$\begin{aligned} & {\tilde{S}}_{-1}^{(k,\alpha)}=\bigl\{ v\in K: \operatorname{min}\bigl\{ A^{(k)}\bigl(v-u_{\alpha }^{(k-1)}\bigr)+r \bigl(u_{\alpha}^{(k-1)}\bigr),v-\phi\bigr\} \leq0\bigr\} . \end{aligned}$$

Moreover, the solutions $u_{\alpha}^{(k)}$ of problems (3.1), $\alpha=1, -1$, are the minimal and maximal elements of $S_{1}^{(k,\alpha)} $ and ${\tilde{S}}_{-1}^{(k,\alpha)}$, respectively.

### Lemma 3.1

*Let* $u_{\alpha}^{(k)}$, $\alpha=1,-1$, *be the solutions of problems* (3.1), *respectively*. *If* $u^{(k-1)}_{1}\ge u^{(k-1)}_{-1}$, $u_{1}^{(k)}\geq u_{-1}^{(k)}$.

### Proof

For any $v\in R^{n}$, we have $v=v^{+}+v^{-}$, where $v^{+}=\max\{v,0\}\geq0$ and $v^{-}=\operatorname{min}\{ v,0\}\leq0$. Let $z^{(k)}=u_{-1}^{(k)}-u_{1}^{(k)}$. It is sufficient to prove that $(z^{(k)})^{+}=0$. By (3.1) and $(z^{(k)})^{+}\ge0$, we have

3.6 $$ \bigl(\bigl(z^{(k)}\bigr)^{+}\bigr)^{T} \bigl[A^{(k)}\bigl(u_{1}^{(k)}-u_{1}^{(k-1)} \bigr)+r\bigl(u_{1}^{(k-1)}\bigr)\bigr]\geq0. $$

If $((z^{(k)})^{+})_{i}>0$, $(u_{-1}^{(k)})_{i}>(u_{1}^{(k)})_{i}\ge\phi_{i}$. Then, by (3.1), we have

$$\bigl[A^{(k)}\bigl(u_{-1}^{(k)}-u_{-1}^{(k-1)} \bigr)+r\bigl(u_{-1}^{(k-1)}\bigr)\bigr]_{i}=0. $$

Thereby,

$$\begin{aligned} &\bigl(\bigl(z^{(k)}\bigr)^{+}\bigr)^{T}\bigl[ A^{(k)} \bigl(u_{-1}^{(k)}-u_{-1}^{(k-1)}\bigr)+r \bigl(u_{-1}^{(k-1)}\bigr)\bigr] \\ &\quad=\sum_{i=1}^{n}\bigl(\bigl(z^{(k)} \bigr)^{+}\bigr)_{i}\bigl[ A^{(k)}\bigl(u_{-1}^{(k)}-u_{-1}^{(k-1)} \bigr)+r\bigl(u_{-1}^{(k-1)}\bigr)\bigr]_{i} \\ &\quad =0. \end{aligned}$$

This together with (3.6) gives

$$\bigl(\bigl(z^{(k)}\bigr)^{+}\bigr)^{T}\bigl[A^{(k)} \bigl(u_{1}^{(k)}-u_{1}^{(k-1)}\bigr)+r \bigl(u_{1}^{(k-1)}\bigr)-A^{(k)}\bigl(u_{-1}^{(k)}-u_{-1}^{(k-1)} \bigr)-r\bigl(u_{-1}^{(k-1)}\bigr)\bigr]\geq 0. $$

By the use of (3.2), we have then

$$\bigl(\bigl(z^{(k)}\bigr)^{+}\bigr)^{T}\bigl[-A^{(k)}z^{(k)}+ \Lambda ^{(k-1)}\bigl(u_{-1}^{(k-1)}-u_{1}^{(k-1)} \bigr)+f\bigl(u_{1}^{(k-1)}\bigr)-f\bigl(u_{-1}^{(k-1)} \bigr)\bigr]\geq0. $$

Hence,

3.7 $$\begin{aligned} &\bigl(\bigl(z^{(k)}\bigr)^{+} \bigr)^{T}Az^{(k)} \\ &\quad\leq \bigl(\bigl(z^{(k)}\bigr)^{+}\bigr)^{T}A^{(k)}z^{(k)} \\ &\quad\leq \bigl(\bigl(z^{(k)}\bigr)^{+}\bigr)^{T}\bigl[ \Lambda ^{(k-1)}z^{(k-1)}+f\bigl(u_{1}^{(k-1)}\bigr)-f \bigl(u_{-1}^{(k-1)}\bigr)\bigr] \\ &\quad= \sum_{i=1}^{n}\bigl( \bigl(z^{(k)}\bigr)^{+}\bigr)_{i}\biggl[ \lambda^{(k-1)}_{i}- \int_{0}^{1}f'_{i}\bigl(\eta ^{(k-1)}_{i}(t)\bigr)\,dt\biggr]\bigl(z^{(k-1)} \bigr)_{i}, \end{aligned}$$

where

$$\eta^{(k-1)}(t)=u_{-1}^{(k-1)}+t\bigl(u_{1}^{(k-1)}-u_{-1}^{(k-1)} \bigr). $$

Since $u^{(k-1)}_{1}\ge u^{(k-1)}_{-1}$, we have then

$$z^{(k-1)}\leq0 \quad\mbox{and}\quad u_{-1}^{(k-1)}\leq \eta^{(k-1)}(t)\leq u_{1}^{(k-1)}. $$

This together with (3.3) implies that the right-hand side of the equality in (3.7) is non-positive, and then

$$0\ge\bigl(\bigl(z^{(k)}\bigr)^{+}\bigr)^{T}Az^{(k)} = \bigl(\bigl(z^{(k)}\bigr)^{+}\bigr)^{T}A\bigl(z^{(k)} \bigr)^{+}+\bigl(\bigl(z^{(k)}\bigr)^{+}\bigr)^{T}A \bigl(z^{(k)}\bigr)^{-}, $$

which implies

$$\begin{aligned} \bigl(\bigl(z^{(k)}\bigr)^{+}\bigr)^{T}A\bigl(z^{(k)} \bigr)^{+}&\leq-\bigl(\bigl(z^{(k)}\bigr)^{+}\bigr)^{T}A \bigl(z^{(k)}\bigr)^{-} \\ &=-\sum_{i,j=1}^{n}a_{ij}\bigl( \bigl(z^{(k)}\bigr)^{+}\bigr)_{i}\bigl(\bigl(z^{(k)} \bigr)^{-}\bigr)_{j} \\ &=-\sum_{i\neq j}a_{ij}\bigl( \bigl(z^{(k)}\bigr)^{+}\bigr)_{i}\bigl(\bigl(z^{(k)} \bigr)^{-}\bigr)_{j} \\ &\leq0, \end{aligned}$$

where the last equality is from $((z^{(k)})^{+})_{i}((z^{(k)})^{-})_{i}=0$, and the last inequality is from $((z^{(k)})^{+})_{i}((z^{(k)})^{-})_{j}\leq0$ and $a_{ij}\le0$ for $i\neq j$. By the positive definite assumption of matrix *A*, we conclude $(z^{(k)})^{+}=0$, and the proof is then complete. □

The following theorem gives the monotone convergence of Algorithm 3.1.

### Theorem 3.1

*Algorithm* 3.1 *is well defined and the sequences* $\{ u_{\alpha}^{(k)}\}$, $\alpha=1,-1$, *generated by* (3.1), *converge monotonically to the unique solution* *u* *of problem* (1.2):

3.8 $$ u_{-1}^{(k-1)}\leq u_{-1}^{(k)} \leq\cdots\leq u\leq\cdots\leq u_{1}^{(k)}\leq u_{1}^{(k-1)}. $$

*Moreover*, $\{u^{(k)}_{1}\}\subset S_{1}$, $\{u^{(k)}_{-1}\}\subset{\tilde{S}}_{-1}$.

### Proof

It follows immediately from $u_{-1}^{(0)}\in{\tilde{S}}_{-1}$ and $u_{1}^{(0)}\in S_{1}$ that

$$u_{-1}^{(0)}\leq u\leq u_{1}^{(0)}. $$

Assume that $u^{(k-1)}_{1}\in S_{1}$ and $u^{(k-1)}_{-1}\in{\tilde{S}}_{-1}$. Then, by (3.1), (3.2) and (3.4), we have that

$$A^{(k)}\bigl(u_{1}^{(k-1)}-u_{1}^{(k-1)} \bigr)+r\bigl(u_{1}^{(k-1)}\bigr)=Au_{1}^{(k-1)}+f \bigl(u_{1}^{(k-1)}\bigr)\ge 0, $$

and for indices *i* satisfying $(u_{-1}^{(k-1)})_{i}>\phi_{i}$,

$$\bigl[A^{(k)}\bigl(u_{-1}^{(k-1)}-u_{-1}^{(k-1)} \bigr)+r\bigl(u_{-1}^{(k-1)}\bigr)\bigr]_{i}= \bigl[Au_{-1}^{(k-1)}+f\bigl(u_{-1}^{(k-1)}\bigr) \bigr]_{i}\leq 0. $$

That is, $u^{(k-1)}_{1}\in S_{1}^{(k,1)}$ and $u^{(k-1)}_{-1}\in{\tilde{S}}_{-1}^{(k,-1)}$, where $S_{1}^{(k,1)}$ and ${\tilde{S}}_{-1}^{(k,-1)}$ are defined by (3.4) and (3.5), respectively. Therefore, by Remark 3.1, we have

3.9 $$ u^{(k)}_{1}\leq u^{(k-1)}_{1}\quad \mbox{and}\quad u^{(k)}_{-1}\geq u^{(k-1)}_{-1}. $$

Let $z_{\alpha}^{(k)}=u_{\alpha}^{(k)}-u_{\alpha}^{(k-1)}$, $\alpha=1,-1$. (3.9) implies

3.10 $$ z_{-1}^{(k)}\geq0\geq z_{1}^{(k)}. $$

From (3.1), we have

3.11 $$\begin{aligned} &Au_{\alpha}^{(k)}+f \bigl(u_{\alpha}^{(k)}\bigr) \\ &\quad= A^{(k)}\bigl(u_{\alpha}^{(k)}-u_{\alpha}^{(k-1)} \bigr)+r\bigl(u_{\alpha }^{(k-1)}\bigr)+f\bigl(u_{\alpha}^{(k)} \bigr)-f\bigl(u_{\alpha}^{(k-1)}\bigr)-\Lambda^{(k-1)}z_{\alpha}^{(k)} \\ &\quad= A^{(k)}\bigl(u_{\alpha}^{(k)}-u_{\alpha}^{(k-1)} \bigr)+r\bigl(u_{\alpha}^{(k-1)}\bigr) \\ &\qquad{} + \int_{0}^{1}\bigl[f' \bigl(u_{\alpha}^{(k-1)}+tz_{\alpha}^{(k)}\bigr)- \Lambda ^{(k-1)}\bigr]z_{\alpha}^{(k)}\,dt, \end{aligned}$$

where

$$f'\bigl(u_{\alpha}^{(k-1)}+tz_{\alpha}^{(k)} \bigr)=\operatorname{diag} \bigl(f'_{i}\bigl(\bigl(u_{\alpha}^{(k-1)}\bigr)_{i}+t\bigl(z_{\alpha}^{(k)} \bigr)_{i}\bigr)\bigr). $$

By Lemma 3.1 and (3.9), for $t\in[0,1]$, we have

$$u_{-1}^{(k-1)}\leq u_{-1}^{(k)}\leq u_{1}^{(k)}\leq u_{1}^{(k-1)}+tz_{1}^{(k)} \le u_{1}^{(k-1)} $$

and

$$u_{-1}^{(k-1)}\leq u_{-1}^{(k-1)}+tz_{-1}^{(k)} \le u_{-1}^{(k)}\leq u_{1}^{(k)}\leq u_{1}^{(k-1)}. $$

And then, by (3.3), we get

3.12 $$ f'_{i}\bigl(\bigl(u_{\alpha}^{(k-1)} \bigr)_{i}+t\bigl(z_{\alpha}^{(k)}\bigr)_{i} \bigr)\leq\lambda_{i}, \quad \forall i=1,2,\ldots, n. $$

It then follows from (3.1) and (3.10)-(3.12) that

$$Au_{1}^{(k)}+f\bigl(u_{1}^{(k)}\bigr)\ge A^{(k)}\bigl(u_{1}^{(k)}-u_{1}^{(k-1)} \bigr)+r\bigl(u_{1}^{(k-1)}\bigr)\ge0, $$

which implies $u_{1}^{(k)}\in S_{1}$. On the other hand, if $(u_{-1}^{(k)})_{i}>\phi_{i}$, we have by (3.1) and (3.10)-(3.12) that

$$\bigl[Au_{-1}^{(k)}+f\bigl(u_{-1}^{(k)} \bigr)\bigr]_{i} = \int_{0}^{1}\bigl[f'_{i} \bigl(\bigl(u_{-1}^{(k-1)}\bigr)_{i}+t \bigl(z_{-1}^{(k)}\bigr)_{i}\bigr)-\lambda _{i}\bigr]\bigl(z_{-1}^{(k)}\bigr)_{i}\,dt \le0, $$

which implies $u_{-1}^{(k)}\in{\tilde{S}}_{-1}$. By the principle of induction, we obtain (3.8) as well as $\{u^{(k)}_{1}\}\subset S_{1}$ and $\{u^{(k)}_{-1}\}\subset {\tilde{S}}_{-1}$. Furthermore, by (3.8), sequences $u^{(k)}_{1}$ and $u^{(k)}_{-1}$ are monotone and bounded. Therefore, they are convergent. Let $\lim u_{\alpha}^{(k)}=u_{\alpha}$, $\alpha=1,-1$. By (3.1), we have $u_{\alpha}\ge\phi$ and

$$r(u_{\alpha})\ge0,\quad (u_{\alpha}-\phi)^{T}r(u_{\alpha})=0, $$

which implies $u_{1}=u_{-1}=u$ by (3.2). We then complete the proof. □

## Quadratic convergence rate of the monotone algorithm

Introduce the notations

4.1 $$ c_{1}=\operatorname{min}_{i}\bigl\{ \operatorname{min}\bigl\{ f'_{i}(v_{i}):u_{-1}^{(0)} \leq v_{i}\leq u_{1}^{(0)}\bigr\} \bigr\} \geq \operatorname{min}_{i}c_{i}^{*} $$

and

4.2 $$ c_{2}=\max_{i}\bigl\{ \max\bigl\{ \bigl\vert f''_{i}(v_{i}) \bigr\vert :u_{-1}^{(0)}\leq v_{i}\leq u_{1}^{(0)} \bigr\} \bigr\} . $$

Here, we have assumed that *f* is twice continuously differentiable. The following theorem gives the quadratic convergence of Algorithm 3.1.

### Theorem 4.1

*Let the sequences* $\{u_{\alpha}^{(k)}\}$, $\alpha=1,-1$, *be generated by Algorithm* 3.1. *Then the following estimate holds*:

4.3 $$ \bigl\Vert u_{1}^{(k)}-u_{-1}^{(k)} \bigr\Vert \leq\frac{c_{2}}{\sigma+c_{1}} \bigl\Vert u_{1}^{(k-1)}-u_{-1}^{(k-1)} \bigr\Vert ^{2}, $$

*where* $\Vert v \Vert =\sqrt{v^{T}v}$.

### Proof

The linear complementarity problems (3.1) can be reformulated as the following variational inequality problems of finding $u_{\alpha}^{(k)}\in K$, $\alpha=1,-1$, respectively, such that

$$\bigl(A^{(k)}\bigl(u_{\alpha}^{(k)}-u_{\alpha}^{(k-1)} \bigr)+r\bigl(u_{\alpha }^{(k-1)}\bigr),v_{\alpha}-u_{\alpha}^{(k)} \bigr)\geq0,\quad \forall v_{\alpha}\in K, $$

where $(v,w)=v^{T}w$. Letting $v_{1}=u_{-1}^{(k)}$ and $v_{-1}=u_{1}^{(k)}$ in above variational forms, we get

$$\bigl(A^{(k)}\bigl(u_{1}^{(k)}-u_{1}^{(k-1)} \bigr)+r\bigl(u_{1}^{(k-1)}\bigr),u^{(k)}_{-1}-u_{1}^{(k)} \bigr)\geq0 $$

and

$$\bigl(A^{(k)}\bigl(u_{-1}^{(k)}-u_{-1}^{(k-1)} \bigr)+r\bigl(u_{-1}^{(k-1)}\bigr),u^{(k)}_{1}-u_{-1}^{(k)} \bigr)\geq 0. $$

Denoting $u_{-1}^{(k)}-u_{1}^{(k)}$ by $z^{(k)}$ and adding above two inequalities, we obtain

$$\bigl(-A^{(k)}z^{(k)}+A^{(k)}z^{(k-1)}+r \bigl(u_{1}^{(k-1)}\bigr)-r\bigl(u_{-1}^{(k-1)} \bigr),z^{(k)}\bigr)\geq 0. $$

Then, by (3.2), we have

$$\bigl(-A^{(k)}z^{(k)}+\Lambda ^{(k-1)}z^{(k-1)}+f \bigl(u_{1}^{(k-1)}\bigr)-f\bigl(u_{-1}^{(k-1)} \bigr),z^{(k)}\bigr)\geq0. $$

That is,

4.4 $$ \bigl(A^{(k)}z^{(k)},z^{(k)}\bigr)\leq \bigl(\Lambda ^{(k-1)}z^{(k-1)}+f\bigl(u_{1}^{(k-1)} \bigr)-f\bigl(u_{-1}^{(k-1)}\bigr),z^{(k)}\bigr). $$

Taking into account (3.3), we conclude that

4.5 $$\begin{aligned} &\bigl(\Lambda^{(k-1)}z^{(k-1)}+f \bigl(u_{1}^{(k-1)}\bigr)-f\bigl(u_{-1}^{(k-1)} \bigr),z^{(k)}\bigr) \\ &\quad=\biggl(\operatorname{diag}\biggl(\lambda^{(k-1)}_{i}- \int_{0}^{1}f'_{i}\bigl(\xi _{i}^{(k-1)}(t)\bigr)\,dt\biggr)z^{(k-1)},z^{(k)} \biggr) \\ &\quad=\biggl(\operatorname{diag}\biggl( \int_{0}^{1}\bigl[f'_{i} \bigl(\xi_{i}^{(k-1)}\bigl(s_{i}^{(k-1)}\bigr) \bigr)-f'_{i}\bigl(\xi _{i}^{(k-1)}(t) \bigr)\bigr]\,dt\biggr)z^{(k-1)},z^{(k)}\biggr), \end{aligned}$$

where

$$\xi^{(k-1)}(t)=u_{-1}^{(k-1)}+t\bigl(u_{1}^{(k-1)}-u_{-1}^{(k-1)} \bigr) $$

and

$$s_{i}^{(k-1)}\in[0,1],\quad i=1,2,\ldots,n. $$

Therefore, by (4.2),

$$\begin{aligned} & \biggl\vert \int_{0}^{1}\bigl[f'_{i} \bigl(\xi_{i}^{(k-1)}\bigl(s_{i}^{(k-1)}\bigr) \bigr)-f'_{i}\bigl(\xi _{i}^{(k-1)}(t) \bigr)\bigr]\,dt \biggr\vert \\ &\quad \leq c_{2} \int_{0}^{1} \bigl\vert s_{i}^{(k-1)}-t \bigr\vert \,dt \bigl\vert z_{i}^{(k-1)} \bigr\vert \\ &\quad \leq c_{2} \bigl\vert z_{i}^{(k-1)} \bigr\vert . \end{aligned}$$

This together with (1.4), (4.1) as well as (4.4) and (4.5) implies

$$(\sigma+c_{1})\bigl\Vert z^{(k)}\bigr\Vert ^{2}\leq \bigl(A^{(k)}z^{(k)},z^{(k)}\bigr)\leq c_{2} \bigl\Vert z^{(k-1)}\bigr\Vert ^{2}\bigl\Vert z^{(k)}\bigr\Vert . $$

That is, (4.3) holds. The proof is then completed. □

By estimate (4.3), the quadratic convergence holds true for the difference $u_{1}^{(k)}-u_{-1}^{(k)}$ between the upper and lower iterative solutions. This is not like the usual quadratic convergence estimate. Nevertheless, similar to the proof of Theorem 4.1, we can get

$$\bigl(A^{(k)}\bigl(u_{\alpha}^{(k)}-u_{\alpha}^{(k-1)} \bigr)+r\bigl(u_{\alpha }^{(k-1)}\bigr),u-u_{\alpha}^{(k)} \bigr)\geq0 $$

and

$$\bigl(Au+f(u),u_{\alpha}^{(k)}-u\bigr)\geq0. $$

And then,

$$\bigl(-A^{(k)}\bigl(u-u_{\alpha}^{(k)}\bigr)+ \Lambda^{(k-1)}\bigl(u-u_{\alpha }^{(k-1)}\bigr)+f \bigl(u_{\alpha}^{(k-1)}\bigr)-f(u),u-u_{\alpha}^{(k)} \bigr)\geq0. $$

Noting that $u_{-1}^{(k)}\leq u\leq u_{1}^{(k)}$, we can obtain the following standard estimates in a similar way.

### Theorem 4.2

*Let the sequences* $\{u_{\alpha}^{(k)}\}$, $\alpha=1,-1$, *be generated by* (3.1). *Then the following estimates hold*:

4.6 $$ \bigl\Vert u_{1}^{(k)}-u \bigr\Vert \leq \frac{c_{2}}{\sigma+c_{1}} \bigl\Vert u_{1}^{(k-1)}-u \bigr\Vert ^{2}, $$

*if* $f''_{i}(v_{i})\geq0$ *for* $u_{-1}^{(0)}\leq v_{i}\leq u_{1}^{(0)}$, *and*

4.7 $$ \bigl\Vert u_{-1}^{(k)}-u \bigr\Vert \leq \frac{c_{2}}{\sigma+c_{1}} \bigl\Vert u_{-1}^{(k-1)}-u \bigr\Vert ^{2}, $$

*if* $f''_{i}(v_{i})\leq0$ *for* $u_{-1}^{(0)}\leq v_{i}\leq u_{1}^{(0)}$.

Estimates (4.6) and (4.7) indicate that when $f_{i}(v_{i})$ ($i=1,2,\ldots, n$) have convex (or concavity ) property in a neighborhood of the solution of problem (1.2), the maximal (or minimal) sequence, generated by (3.1), converges quadratically to the solution of the problem.

## Extensions to a two-sided obstacle problem

In this section, we extend the results obtained in the previous sections to the case of a two-sided obstacle problem. Consider the discrete two-sided obstacle problem of finding $u\in K$ such that

5.1 $$ \textstyle\begin{cases} (Au+f(u))_{i}\geq0&\mbox{if } u_{i}=\phi_{i},\\ (Au+f(u))_{i}= 0&\mbox{if } \phi_{i}< u_{i}< \psi_{i},\\ (Au+f(u))_{i}\leq0&\mbox{if } u_{i}=\psi_{i}, \end{cases} $$

where *A* and *f* are defined as before, $K=\{v\in R^{n}:\phi\leq v\leq\psi\}$ with $\phi<\psi$.

Problem (5.1) is equivalent to the following system of nonsmooth equations:

5.2 $$ \max\bigl\{ \operatorname{min}\bigl\{ Au+f(u),u-\phi\bigr\} ,u-\psi\bigr\} =0 $$

and the following variational inequality problem of finding $u\in K$ such that

5.3 $$ \bigl(Au+f(u),v-u\bigr)\geq0,\quad \forall v\in K. $$

According to (5.2), we define the upper and lower solution sets of problem (5.1), respectively, as follows:

$$S_{1}=\bigl\{ v\in K: \max\bigl\{ v-\psi,\operatorname{min}\bigl\{ Av+f(v),v-\phi\bigr\} \bigr\} \geq0\bigr\} , $$

which is equivalent to

5.4 $$ S_{1}=\bigl\{ v\in K: \bigl(Av+f(v)\bigr)_{i} \ge0, \mbox{ if } v_{i}< \psi_{i}\bigr\} , $$

and

$$S_{-1}=\bigl\{ v\in K: \max\bigl\{ v-\psi,\operatorname{min}\bigl\{ Av+f(v),v-\phi\bigr\} \bigr\} \leq0\bigr\} , $$

which is equivalent to

5.5 $$ S_{-1}=\bigl\{ v\in K: \bigl(Av+f(v)\bigr)_{i} \leq0, \mbox{ if } v_{i}>\phi_{i}\bigr\} . $$

Similar to the case of complementarity problem, the solution of problem (5.1) is the minimal (maximal) element of $S_{1}$ ($S_{-1}$).

Obviously, *ϕ* and *ψ* are candidates of $S_{1}$ and $S_{-1}$, respectively. In the following, we present two schemes, which can produce an upper or a lower solution of problem (5.1) from any $w\in R^{n}$.

### Scheme 5.1

Let $w\in R^{n}$.

*Step* 1.: Solve the following LCP of finding $\bar{u}\ge\phi$ such that
  5.6 $$ \bigl(A+\Lambda^{*}\bigr)\bar{u}\ge\bar{g}, \qquad (\bar{u}- \phi)^{T}\bigl[\bigl(A+\Lambda^{*}\bigr)\bar{u}-\bar{g}\bigr]=0, $$
  where $\Lambda^{*}$ and *ḡ* are the same as those given in Section 2.
*Step* 2.: Let
  $$\tau_{i}= \textstyle\begin{cases} 0 & \mbox{if } \bar{u}_{i}\leq\psi_{i},\\ \psi_{i}-\bar{u}_{i} & \mbox{if } \bar{u}_{i}> \psi_{i} \end{cases} $$
  and $\Gamma=\operatorname{diag}(\tau_{1},\tau_{2},\ldots,\tau_{n})$. Define
  $$\tilde{\bar{u}}=\bar{u}+\Gamma e, $$
  where $e\in R^{n}$ is the vector of ones.

By Scheme 5.1, $\tilde{\bar{u}}\leq\bar{u}$ and $\tilde{\bar{u}}\in K$. Moreover, if $\tilde{\bar{u}}_{i}<\psi_{i}$,

$$\bigl(A\tilde{\bar{u}}+f(\tilde{\bar{u}})\bigr)_{i}=\bigl(A\bar{u}+f( \bar{u})\bigr)_{i}+\sum_{j=1}^{n}a_{ij} \tau_{j} +f_{i}(\tilde{\bar{u}} _{i})-f_{i}( \bar{u}_{i}) \ge\sum_{j\neq i}a_{ij} \tau_{j}\ge0, $$

where the first inequality is from Lemma 2.1, $\tau_{i}=0$ and $\bar{u}_{i}=\tilde{\bar{u}}_{i}$, the second inequality is from the definition of *M*-matrix and $\tau_{j}\leq0$ for each *j*.

Thereby, we obtain the following result.

### Lemma 5.1

*Let* $\tilde{\bar{u}}$ *be produced by Scheme* 5.1. *Then* $\tilde{\bar{u}}\in S_{1}$, *where* $S_{1}$ *is defined by* (5.4).

Similarly, we can obtain a lower solution of the problem by the following scheme.

### Scheme 5.2

Let $w\in R^{n}$.

*Step* 1.: Solve the following LCP of finding $\underline{u}\leq\psi$ such that
  5.7 $$ \bigl(A+\Lambda^{*}\bigr)\underline{u}\leq\underline{g},\qquad (\underline{u}-\psi )^{T}\bigl[\bigl(A+\Lambda^{*}\bigr)\underline{u}-\bar{g}\bigr]=0, $$
  where $\Lambda^{*}$ and $\underline{g}$ are the same as those given in Section 2.
*Step* 2.: Let
  $$\tau_{i}= \textstyle\begin{cases} 0 & \mbox{if } \bar{u}_{i}\geq\phi_{i},\\ \phi_{i}-\underline{u}_{i} & \mbox{if } \bar{u}_{i}< \phi_{i} \end{cases} $$
  and $\Gamma=\operatorname{diag}(\tau_{1},\tau_{2},\ldots,\tau_{n})$. Define
  $$\tilde{\underline{u}}=\underline{u}+\Gamma e. $$

Similar to Lemma 5.1, we have the following result.

### Lemma 5.2

*Let* $\tilde{\underline{u}}$ *be produced by Scheme* 5.2. *Then* $\tilde{\bar{u}}\in S_{-1}$, *where* $S_{-1}$ *is defined by* (5.5).

By Schemes 5.1 and 5.2, we can obtain a pair of upper and lower solutions of two-sided obstacle problem (5.1) by solving two affine upper and lower obstacle problems (5.6) and (5.7), instead of solving one two-sided obstacle problem. To our knowledge, as for the two-sided obstacle problem, direct algorithms with polynomial computational complexity are few.

### Algorithm 5.1

Let the initials $u^{(0)}_{1}\in S_{1}$ and $u^{(0)}_{-1}\in{ S}_{-1}$. For $k\ge1$, we calculate a pair of iterates by solving the following affine two-sided obstacle problems of finding $u_{\alpha}^{(k)}\in K$, $\alpha=1, -1$, respectively, such that

5.8 $$ \textstyle\begin{cases} [A^{(k)}(u_{\alpha}^{(k)}-u_{\alpha}^{(k-1)})+r(u_{\alpha }^{(k-1)})]_{i}\ge0&\mbox{if }( u_{\alpha}^{(k)})_{i}=\phi_{i},\\ [A^{(k)}(u_{\alpha}^{(k)}-u_{\alpha}^{(k-1)})+r(u_{\alpha}^{(k-1)})]_{i}= 0&\mbox{if } \phi_{i}< (u_{\alpha}^{(k)})_{i}< \psi_{i},\\ [A^{(k)}(u_{\alpha}^{(k)}-u_{\alpha}^{(k-1)})+r(u_{\alpha }^{(k-1)})]_{i}\leq0&\mbox{if } (u_{\alpha}^{(k)})_{i}=\psi_{i}, \end{cases} $$

where matrix $A^{(k)}$ and mapping *r* are the same as those in Algorithm 3.1.

Similar to Lemma 3.1, the following lemma holds.

### Lemma 5.3

*Let* $u_{\alpha}^{(k)}$, $\alpha=1,-1$, *be the solutions of problems* (5.1), *respectively*. *If* $u^{(k-1)}_{1}\ge u^{(k-1)}_{-1}$, $u_{1}^{(k)}\geq u_{-1}^{(k)}$.

According to Lemma 5.3, we have the following monotone and quadratic convergence similar to Theorems 3.1, 4.1 and 4.2.

### Theorem 5.1

*Let the sequences* $\{u_{\alpha}^{(k)}\}$, $\alpha=1,-1$, *be generated by Algorithm* 5.1. *Then the iterates converge to the solution* *u* *of problem* (5.1). *Moreover*, $\{u^{(k)}_{1}\}\subset S_{1}$, $\{u^{(k)}_{-1}\}\subset S_{-1}$, *and*

$$\begin{aligned} &u_{-1}^{(k-1)}\leq u_{-1}^{(k)}\leq\cdots\leq u\leq\cdots\leq u_{1}^{(k)}\leq u_{1}^{(k-1)}, \\ &\bigl\Vert u_{1}^{(k)}-u_{-1}^{(k)} \bigr\Vert \leq\frac{c_{2}}{\sigma+c_{1}} \bigl\Vert u_{1}^{(k-1)}-u_{-1}^{(k-1)} \bigr\Vert ^{2}, \end{aligned}$$

*where the constants are the same as those in Theorem* 4.1. *Moreover*,

$$\bigl\Vert u_{1}^{(k)}-u \bigr\Vert \leq \frac{c_{2}}{\sigma+c_{1}} \bigl\Vert u_{1}^{(k-1)}-u \bigr\Vert ^{2}, $$

*if* $f''_{i}(v_{i})\geq0$ *for* $u_{-1}^{(0)}\leq v_{i}\leq u_{1}^{(0)}$, *and*

$$\bigl\Vert u_{-1}^{(k)}-u \bigr\Vert \leq\frac{c_{2}}{\sigma+c_{1}} \bigl\Vert u_{-1}^{(k-1)}-u \bigr\Vert ^{2}, $$

*if* $f''_{i}(v_{i})\leq0$ *for* $u_{-1}^{(0)}\leq v_{i}\leq u_{1}^{(0)}$.

## Numerical experiments

In this section, we present numerical experiments in order to investigate the performance of the proposed algorithms. The programs are coded in Visual C++ 6.0 and run on a computer with 2.0 GHz CPU. We consider the following two problems.

### Problem 1

We consider the following nonlinear complementarity problem which is the same as Problem 5.1 in [20]:

$$u\ge0, F(u)\ge0, \quad u^{T}F(u)=0. $$

Here $F(u)=Au+D(u)+f$, where

$$A= \frac{1}{h^{2}} \left( \begin{matrix} H&-I&&\\ -I&H&\ddots&\\ &\ddots&\ddots&-I\\ &&-I&H \end{matrix} \right) $$

and

$$H= \left( \begin{matrix} 4&-1&&\\ -1&4&\ddots&\\ &\ddots&\ddots&-1\\ &&-1&4 \end{matrix} \right), $$

$h=\frac{1}{\sqrt{n}+1}$, $D(x)=(D_{i}):R^{n}\rightarrow R^{n}$ being a given diagonal mapping with $D_{i}:R\rightarrow R$, for $i=1,2,\ldots,n$, that is, component $D_{i}$ of *D* is a function of the *i*th variable $u_{i}$ only. Set $D_{i}(u_{i})=\lambda e^{u_{i}}$ and obtain a diagonal mapping $D(u)=(D_{i}(u_{i}))$. In our test, we fix $\lambda=0.8$, and let $f_{i}=\max\{ 0,v_{i}-0.5\}\times10^{w_{i}-0.5}$, where $w_{i}$ and $v_{i}$ are random numbers in $[0,1]$, $i=1,2,\ldots, n$.

### Problem 2

We discuss the following nonlinear complementarity problem:

$$u\ge0, F(u)\ge0,\quad u^{T}F(u)=0. $$

Here $F(u)=Au+D(u)+f$, with *A* being the same as in Problem 1. The vector *f* is generated from a uniform distribution in the interval $(-10,10)$. Let $D(u)=(D_{i}):R^{n}\rightarrow R^{n}$ be a given diagonal mapping with $D_{i}:R\rightarrow R$, for $i=1,2,\ldots,n$. The components of $D(x)$ are $D_{i}(u)=\operatorname{arctan}(u_{i})$.

We compare different algorithms from the point of view of iteration numbers and cpu times (seconds). Here, we consider three algorithms: Algorithm 3.1, denoted by (AL); the semismooth equation approach proposed in [21], denoted by SSN; and primal-dual algorithm proposed in [22], denoted by PDA.

In the algorithm AL, we choose initial point $u^{(0)}_{-1}=0$ and $u^{(0)}_{1}$ to be obtained by Lemma 2.1 for all problems. All subproblems are solved by PSOR and the tolerance in PSOR is chosen to be equal to 10^−7^ in $\Vert \cdot \Vert _{2}$ norm. In order to determine the relaxation parameter *ω*, we consider the following nonlinear complementarity problem:

$$u\ge0, F(u)\ge0, \quad u^{T}F(u)=0. $$

Here $F(u)=Au+D(u)+f$, where *A* is the same in Problems 1 and 2, $D(u)=(D_{i}):R^{n}\rightarrow R^{n}$ being a given diagonal mapping like in Problem 1. Set $D_{i}(u_{i})=u_{i}^{2}$, and let $f_{i}=-1$. Fix $h=\frac{1}{20}$, and use AL to solve the above problem. We change the *ω*, and present the result in Table 1. From the table, we can see that the relaxation parameter $\omega=1.9$ is a good choice, and we use this in all problems.

**Table 1:** **Comparisons of** ***ω***

***ω***	**iter**	***ω***	**iter**	***ω***	**iter**
0.9	225	1.1	152	1.3	101
1.5	64	1.7	36	1.9	22

The termination criterion of the algorithm AL is chosen to be $\Vert u^{(k)}_{1}-u^{(k)}_{-1} \Vert \le10^{-6}$. In the algorithm SSN, we choose initial point $u^{0}=0$, tolerance $\epsilon=10^{-6}$, $p=3$, $\rho=0.5$, $\beta=0.3$ and $H_{k}\in\partial_{B} \Phi$ is defined by the procedure proposed in [21]. In the algorithm PDA, we fix $c=1$ and choose the initial point $u^{0}=0$. The stopping criterion is that the active sets are not changed between two iterations. In this algorithm, the subproblems are systems of nonlinear equations, which are solved by Newton iteration. The numerical results are listed in Tables 2-3, where ‘iter’ denotes the iteration number for the algorithm converging to the solution and ‘cpu’ denotes the execution time.

**Table 2:** **Comparisons of iteration numbers and execution times for Problem** **1**

***h***	**AL**		**SSN**		**PDA**	
	**iter**	**cpu**	**iter**	**cpu**	**iter**	**cpu**
$\frac{1}{10}$	2	0.013	3	0.01	2	0.001
$\frac{1}{20}$	2	0.678	3	0.603	2	0.012
$\frac{1}{40}$	2	9.246	3	37.883	2	0.187
$\frac{1}{60}$	2	237.486	3	434.057	2	0.932

**Table 3:** **Comparisons of iteration numbers and execution times for Problem** **2**

***h***	**AL**		**SSN**		**PDA**	
	**iter**	**cpu**	**iter**	**cpu**	**iter**	**cpu**
$\frac{1}{10}$	2	0.1	5	0.019	1	0.001
$\frac{1}{20}$	2	0.775	5	1.011	1	0.037
$\frac{1}{40}$	2	12.768	7	89.629	4	8.653
$\frac{1}{60}$	3	444.401	22	3,177.98	11	1,024.21

From Tables 2-3, we can easily see that the iteration numbers of Algorithm 3.1 are stable, which may also mean that the initials obtained by (2.2) may be a good solution guess. While for SSN and PDA, the iteration numbers increase when the dimensions of Problem 2 become large. As we can see from Table 3, the algorithm we proposed seems to be more effective for solving large-scale problems. The main reason may be as follows. Algorithm 3.1 takes only a few iterations for all problems, and in each iteration it only needs to solve two linear complementarity problems. These subproblems are solved by PSOR rapidly. For SSN, it takes a lot of time to solve the system of linear equations to obtain directions, especially for large-scale problems. For PDA, by using the active set strategy, in each iteration, it only needs to solve a reduced system of linear equations, where the dimension is much less than the original one. Therefore, the execution time for PDA is also less compared with SSN.

## Conclusions

In this paper, we have considered the numerical solution for the discretization of semilinear elliptic complementarity problems. Based on the upper and lower solutions of the problem, we have proposed a monotone algorithm and proved that iterates are a pair of upper and lower solution iterates and converge monotonically from above and below, respectively, to the solution of the problem. Moreover, we have established quadratic convergence of the algorithm. The limited numerical results showed that the proposed algorithm is effective.
